# Elevated Perspectives: Unraveling Cardiovascular Dynamics in High-Altitude Realms

**DOI:** 10.2174/011573403X308818241030051249

**Published:** 2024-11-05

**Authors:** Kanishk Aggarwal, Mayur Srinivas Pathan, Mayank Dhalani, Inder P. Kaur, Fnu Anamika, Vasu Gupta, Dilip Kumar Jayaraman, Rohit Jain

**Affiliations:** 1 Internal Medicine, Dayanand Medical College, Punjab, India;; 2 Avalon University School of Medicine, Willemstad, Curacao;; 3 GMERS Medical College & Hospital, Gotri, Vadodara, Gujrat, India;; 4 University of Mississippi Medical Center, Jackson, Mississippi, MS 39216, USA;; 5 University College of Medical Sciences, New Delhi, India;; 6 Dayanand Medical College, Punjab, India;; 7 Main Line Health, Paoli, PA 19301, USA;; 8 Penn State Milton S. Hershey Medical Center, Hershey, PA 17033, USA

**Keywords:** Cardiovascular disease, congenital heart disease, cardiovascular health, himalayas, pathophysiology, high altitude

## Abstract

High-altitude regions pose distinctive challenges for cardiovascular health because of decreased oxygen levels, reduced barometric pressure, and colder temperatures. Approximately 82 million people live above 2400 meters, while over 100 million people visit these heights annually. Individuals ascending rapidly or those with pre-existing cardiovascular conditions are particularly vulnerable to altitude-related illnesses, including Acute Mountain Sickness (AMS) and Chronic Mountain Sickness (CMS). The cardiovascular system struggles to adapt to hypoxic stress, which can lead to arrhythmias, systemic hypertension, and right ventricular failure. Pathophysiologically, high-altitude exposure triggers immediate increases in cardiac output and heart rate, often due to enhanced sympathetic activity. Over time, acclimatisation involves complex changes, such as reduced stroke volume and increased blood volume. The pulmonary vasculature also undergoes significant alterations, including hypoxic pulmonary vasoconstriction and vascular remodelling, contributing to conditions, like pulmonary hypertension and high-altitude pulmonary edema. Genetic adaptations in populations living at high altitudes, such as gene variations linked to hypoxia response, further influence these physiological processes. Regarding cardiovascular disease risk, stable coronary artery disease patients generally do not face significant adverse outcomes at altitudes up to 3500 meters. However, those with unstable angina or recent cardiac interventions should avoid high-altitude exposure to prevent exacerbation. Remarkably, high-altitude living correlates with reduced cardiovascular mortality rates, possibly due to improved air quality and hypoxia-induced adaptations. Additionally, there is a higher incidence of congenital heart disease among children born at high altitudes, highlighting the profound impact of hypoxia on heart development. Understanding these dynamics is crucial for managing risks and improving health outcomes in high-altitude environments.

## 1 INTRODUCTION

High altitudes, like the Himalayas, Alps, Andes, *etc*., attract increasing numbers of tourists, trekkers, and pilgrims. High-altitude living is the condition of residing at 2400 meters above sea level. Over 82 million individuals reside in these regions worldwide, and over 100 million lowlanders visit these areas annually [1]. Altitude is categorised into three levels: high [8,000-12,000 feet (2,438-3,658 meters)], very high [12,000-18,000 feet (3,658-5,487 meters)], and extremely high [18,000+ feet (5,500+ meters)] [2]. As altitude increases, the climate changes gradually, characterised by lower barometric pressure, lower partial pressure of inspired oxygen, lower ambient temperature, and stronger UV sun radiation [3]. When people travel to high altitudes, their bodies undergo a series of physiological reactions that allow them to gradually adapt to lower ambient oxygen levels over several days to weeks. This adaptation process may sometimes result in maladaptive responses, potentially exposing individuals to acute altitude illnesses. The most common altitude sickness is Acute Mountain Sickness (AMS), characterised by non-specific symptoms, such as shortness of breath, headache, tachycardia, *etc*. [4]. Young people under 25 years of age, rapid climbers to an altitude 3500 m above sea level or lowland newcomers, obese, and those with arterial oxygen saturation (SaO_2_) below 80% are primarily at risk of developing AMS [5]. People who inhabit these areas develop mechanisms to adjust to hypobaric hypoxia [6]. Research on populations residing in high altitudes has revealed various phenotypic and genotypic variations that point to varying degrees of adaptation. A study that found three potential genes linked to cardiac function, BRINP3, NOS2, and TBX5, indicated that natural selection may target the cardiovascular system to counteract the substantial stress on the pulmonary and vascular systems brought on by living at high elevations [7]. Healthy individuals residing at high altitudes display significant physiological and anatomic features related to the heart and pulmonary circulation that are similar to those seen in long-term clinical disorders linked to polycythemia, hypoxemia, and alveolar hypoxia-like Right Ventricular Hypertrophy (RVH), Pulmonary Hypertension (PH), and increased amount of Smooth Muscle Cells (SMCs) in the distal pulmonary arterial branches. However, when the ability to adjust to high altitudes is lost, chronic mountain sickness occurs in the form of exacerbated polycythemia and intensified hypoxemia, in addition to moderate to severe pulmonary hypertension [8]. The inability of the cardiovascular system to adapt to high altitudes can lead to several other unfavourable consequences, such as an increased risk of arrhythmias, systemic hypertension, RV failure, and so forth [9].

On the contrary, based on the data currently available, living at higher altitudes is also linked to a decreased risk of dying from heart attacks, strokes, and several forms of cancer. The favourable benefits on cardiovascular health may be partially attributed to the moderate hypoxia at altitudes up to 2500 m. Hypoxia-induced Factor (HIF) pathways may act as a mediator in the altitude-induced decrease in cardiovascular mortality. Reducing air pollution with increasing altitude could be another factor contributing to the decline in coronary heart disease mortality [10]. UV radiation levels increase by approximately 10% for every 300 meters of altitude gain, which can substantially affect cardiovascular mortality as higher levels of vitamin D mediate the protective effects of UV light by likely reducing the risk of thrombus formation [11]. This review has delved into the intricate connection among cardiovascular health, diseases, and high-altitude environments. It has explored the rapid and gradual cardiovascular responses to high-altitude exposure, considering both the general population and those with pre-existing heart conditions.

## 2 PATHOPHYSIOLOGY

The interactions among high-altitude environments, individual genetics, lifestyle, and the processes of adaptation and acclimatisation to various altitudes are incredibly complex. This complexity and dilemma make it challenging to make straightforward predictions about the effects of high altitude on cardiovascular physiology and pathology [2].

## 3 EFFECT ON CARDIAC OUTPUT

Exposure to high altitude can cause cardiovascular stress, leading to an immediate increase in cardiac output. This increase is primarily due to a rise in heart rate and possibly increased stroke volume. Some studies suggest that increased stroke volume is due to enhanced sympathetic activity and increased venous return [6]. However, decreased stroke volume during acclimatisation returns cardiac output to normal levels. This decrease in stroke volume is thought to be due to reduced ventricular filling caused by reduced preload because of pulmonary vasoconstriction. In addition to the reduced stroke volume, there is increased blood volume with acclimatisation despite venous return being normalised through neoangiogenesis of tissue. The prevailing theory posits that muscle activity diminishes at high altitudes, curtailing the demand for elevated blood circulation. Alternatively, another hypothesis proposes that hypoxemia triggers a decline in myocardial performance [2, 12].

## 4 EFFECT ON PULMONARY VASCULATURE

At elevated altitudes, hypoxia induces pulmonary vasoconstriction, leading to an increase in pulmonary arterial pressure. This increase typically follows a parabolic pattern, with pressures rising gradually beyond 3000 meters, increasing from a mean of 15 mmHg at 2000 m to 28-11 mmHg at 4540 m [8]. Vasoconstriction is an effective compensatory mechanism when a small portion of the lungs is hypoxic, as with higher altitudes. However, if hypoxia occurs in large parts of the lungs, it can result in pulmonary hypertension [13]. Furthermore, chronic hypoxia induces muscle hypertrophy in distal vessels, along with the extension of this muscular tissue into typically non-vascularized arterioles, representing a distinctive adaptation to prolonged altitude exposure [14]. By elevating the aldosterone level and increasing the influx of growth factors, hypoxia leads to smooth muscle thickening and pulmonary artery remodelling through the recruitment of leukocytes and fibrocytes, which contribute to vascular remodelling by stimulating angiogenesis and producing collagen that promotes fibrosis [15]. The molecular mechanisms underlying the remodelling of pulmonary vasculature have elucidated that chronic hypoxia causes the formation of reactive oxygen species inside the mitochondria, releasing intracellular calcium through calcium and potassium channels. Alveolar hypoxia is one of the factors that can lead to cardiopulmonary dysfunction and pulmonary hypertension. In High Altitude Pulmonary Edema (HAPE), Hypoxic Pulmonary Vasoconstriction (HPV) drives acute pulmonary hypertension. As a result of acute HPV, blood flow is diverted from poorly ventilated lung segments to optimally ventilated segments to improve ventilation-perfusion matching [16-18].

## 5 EFFECT ON HEART RATE

Heart Rate (HR) increase is the foremost primaeval response to high altitude. Imbalance of the autonomic nervous system by increasing sympathetic activity and decreasing parasympathetic system plays an essential role in increasing HR at rest at the initial rise to high altitude [19]. However, with chronic altitude exposure, acclimatisation ensues, resulting in enhanced parasympathetic activity, causing a decrease in maximum HR [20]. Lowland men ascending beyond 3500 meters exhibited increased sympathetic activity, directly stimulating the β-adrenergic receptors in cardiac tissue and increasing cardiac output, effectively sustaining blood pressure and peripheral oxygen delivery [12]. As often seen in previous studies, an increased heart rate due to sympathetic overactivity has been attributed to the pulmonary stretch receptors regulating HR in this environment. However, another theory has been postulated, which states the critical role of Endothelin-1 (ET-1) in regulating HR at altitude [21]. Ascending to high altitudes correlates with increased plasma ET-1 due to hypoxia-induced ET-1 production in blood vessel lining cells [22]. Stimulating endothelin-1- b-type receptors within cardiomyocytes triggers the release of calcium ions from the sarcoplasmic reticulum, increasing heart rate and cardiac contractility. Furthermore, the turnover of cardiomyocyte calcium mediates the influence of β-adrenergic activity, whereby phosphorylation of molecular targets enhances calcium release during systole and facilitates calcium sequestration for diastolic relaxation. β-adrenergic activity also induces the expression of genes encoding calcium-transporting proteins through CREB interaction, which is particularly evident under hypoxia-reoxygenation conditions that promote CREB DNA-binding and the expression of its target genes. Activated CREB further encourages the synthesis of vital factors, such as Bcl-2, sarcoplasmic reticulum calcium ATPase, and sarcolemmal Na^+^/Ca^2+^ exchanger, potentially preserving mitochondrial integrity and ensuring calcium homeostasis during pathological hypoxic conditions triggered by β-adrenergic activation [2, 23, 24].

## 6 EFFECT ON BLOOD PRESSURE

The changes in Blood Pressure (BP) are another manifestation of the cardiovascular system during acute and chronic altitude exposure [12]. Initially, hypoxia induces systemic vasodilation, causing a decrease in systolic BP, which is quickly counteracted by increased sympathetic activity. This increase in sympathetic activity occurs by sensing decreased oxygen concentration in the blood by receptors in the carotid bodies. These receptors relay signals to the cardiovascular control regions of the midbrain *via* the arterial peripheral chemoreceptors located in the carotid bodies, increasing systolic blood pressure [25, 26]. People residing at high altitudes experience chronic exposure to low oxygen levels, leading to systemic vascular constriction and elevated blood pressure. This widespread vascular constriction results from heightened plasma noradrenaline levels and increased red blood cell mass due to enhanced erythropoietin secretion triggered by hypoxia, as well as the involvement of the renin-angiotensin system [12]. Several investigations on the Renin-angiotensin-aldosterone System (RAAS) at high altitudes have revealed an initial decline in plasma levels of renin, aldosterone, and angiotensin II. However, these levels return to normal upon acclimatisation, accompanied by a reduction in the activity of renin and aldosterone [27]. On a molecular level, hypoxia stimulates the production of a protein called ET-1 in the endothelial cells of blood vessels. Exposure to high altitudes has been found to increase the concentration of ET-1 in the blood. However, excessive release of this protein can lead to pulmonary hypertension and pulmonary edema, characterised by high blood pressure and fluid accumulation in the lungs [28]. Angiotensin II, a hormone that regulates blood pressure, has been found to stimulate the release of ET-1 and enhance its vasoconstrictor effect by upregulating the endothelin-1-type A receptor in vascular smooth muscle. On the other hand, through its interactions with the endothelin type B receptor, ET-1 can cause transient vascular dilation, resulting in low blood pressure by releasing nitric oxide and prostanoids. This may explain the brief hypoxic hypotension that occurs upon acute exposure to high altitudes [12].

## 7 GENETICS AND EPIGENETICS EVIDENCE FOR HIGH-ALTITUDE ADAPTATION

Various genome-wide studies, especially involving the population of the Amhara ethnic group Tibetans, Ethiopian, and Han populations, have elucidated that several genes, like CBARA1, VAV3, ARNT2, THRB, and several haplotypes of EGLN1 and PPARA are essential for adaptation at high altitudes [6]. CBARA1 is a gene that regulates the uptake of calcium by the mitochondria. It is believed to be crucial in low oxygen tension in the blood.

**Fig. (1):**
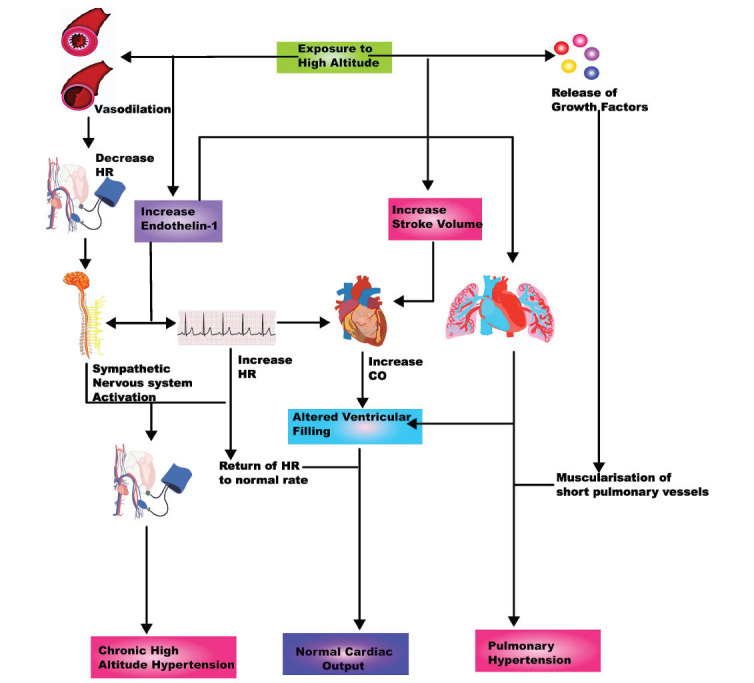
Pathophysiological response to high altitude. **Abbreviations:** HR - heart rate, CO - cardiac output.

VAV3 is another gene that induces GTPase activity and is involved in forming new blood vessels in response to low oxygen tension of blood. The ARNT2 gene is associated with the HIF-1 pathway and forms a heterodimer expressed in the fetal lung. THRB, on the other hand, is needed for HIF expression in hepatic cells, which is the primary source of Erythropoietin (EPO) during fetal development [6]. The PPARA and EPAS1 genes, along with ARNT2 and THRB, are involved in the HIF-1 pathway cascade, are initiated in response to hypoxic environmental conditions, and contain Small Nuclear Proteins (SNPs) with marginal associations with haemoglobin; this suggests that variations at these loci may play a role in adapting to the Ethiopian population's high altitude [29]. The Endothelial PAS domain-containing protein-1 (EPAS1) gene codes for a transcription factor of the Hypoxia-inducible Factor 2α (HIF-2α), which is responsible for stimulating erythrocyte production. Tibetan inhabitants, who have lived in the region for millennia, exhibit normal hemoglobin levels despite experiencing arterial hypoxemia. This resilience is attributed to a genetic variation in the EPAS1 gene, which moderates the erythropoietic response to reduced oxygen saturation, a key feature of altitude adaptation among Tibetans. Variations in the EPAS1 gene have been associated with lower hemoglobin levels, with individuals homozygous for the polymorphism, showing a hemoglobin concentration 0.8 g/dL lower than those who are heterozygous (Fig. **1**) [6, 30].

## 8 DISCUSSION

Investigating potential links between Cardiovascular Disease (CVD) and high altitudes is essential due to the increasing prevalence of these conditions and the large number of people exposed to high altitudes for work or leisure. Acute mountain sickness is the most common illness affecting unacclimatized people exposed to both acute [31] and intermittent [32] hypobaric hypoxia. The extent of this pathology mainly depends upon the individual’s speed of ascent and altitude [31]. The symptoms of AMS (headache, nausea/vomiting, fatigue and/or weakness, and dizziness/lightheadedness) can limit the activity and abilities of people experiencing this condition, especially during the first few days of high-altitude exposure [33]. Chronic Mountain Sickness (CMS) is a medical condition characterised by increased hematocrit levels and excessive polycythemia in patients at high altitudes [34]. CMS is typically characterised by at least three symptoms: breathlessness, palpitations, sleep disturbance, cyanosis, vein dilatation, paresthesia, headache, or tinnitus [35]. In contrast to other pathologies associated with high altitude, CMS is found in residents living at high altitude (exposed to chronic hypobaric hypoxia), which is estimated to include 85 million people [36]; the prevalence of CMS (10–15%) can vary by altitude level, age, and genetic factors [37].

Ascending to high altitude can have adverse effects on individuals with underlying coronary artery disease, congestive heart failure, arrhythmias, systemic hypertension, and respiratory illnesses, particularly in those who ascend rapidly from low altitudes [38]. For cardiac patients, hypoxemia can be harmful even at sea level [26], presenting a significant risk for those with Coronary Artery Disease (CAD) and travelling to higher altitudes. This risk is compounded by increased myocardial oxygen demand due to elevated heart rates, potentially leading to myocardial oxygen deficit and adverse events, including secondary myocardial infarction [39]. However, research suggests that patients with stable CAD do not experience a significant increase in adverse outcomes up to 3500 meters [39]. Conversely, individuals with unstable angina or recent myocardial infarction should avoid physically demanding activities at high altitudes. Patients with acute coronary syndrome, recent angioplasty with stent placement, or recent coronary artery bypass grafting should also refrain from such activities [40].

While the impact of altitude on the prevalence of cardiovascular risk factors may be unclear or variable in existing studies, there is a more precise and consistent association between higher altitudes and reduced cardiovascular mortality risk. A study in Switzerland found that ascending to higher altitudes correlates with lower mortality rates for both men and women with coronary heart disease and stroke, with a 22% reduction in coronary heart disease mortality and a 12% reduction in stroke mortality per 1000-meter increase in altitude [41, 42]. Similarly, a research study in Austria indicated that individuals living at altitudes of 1000-2000 meters had a lower risk of coronary artery disease mortality, with a 31% reduction for women and 28% for men [42]. These findings align with studies showing longer life expectancies in U.S. counties with average elevations above 1500 meters compared to those at sea level, with increases ranging from 0.5 to 2.5 years for women and 1.2 to 3.6 years for men [42].

In addition to coronary heart disease, the incidence of congenital heart disease is much higher in newborns born at high altitudes than those born at lower altitudes. This is particularly true for left-to-right shunt defects, though complex congenital heart disease can also occur. Infants aged 12-18 months at high altitudes have a congenital heart disease incidence about ten times higher than those at lower altitudes, with approximately 8% developing Pulmonary Arterial Hypertension (PAH) or dying [43]. In Nagqu, Tibet, Chun *et al*. investigated 84302 students and found a congenital heart disease prevalence rate of 0.5%, meaning one congenital heart disease among every 200 individuals. The most common defects were Patent Ductus Arteriosus (PDA) (about two-thirds of cases), Atrial Septal Defects (ASDs) (about one-fifth of cases), and Ventricular Septal Defects (VSDs) (about one-tenth of cases), with girls having higher prevalence than boys for congenital heart disease [44]. According to a study conducted in Qinghai province, the prevalence of congenital heart disease among children aged 3-19 years was 6.73%, and it increased with altitude. The prevalence of congenital heart disease was significantly higher among females, those living at higher altitudes, and the Han population [45]. Cross-sectional studies in Tibet between 2012-2013 found that congenital heart disease prevalence varied with altitude, ranging from 4.3% to 4.6% at lower altitudes (≤ 4200 m) and from 12.1% to 13.4% at higher altitudes (> 4200 m) [46, 47].

Patients with underlying heart disease may also experience increased rates of supraventricular and ventricular arrhythmias when rapidly ascending to high altitudes [48]. Kujaník *et al*. found the incidence of Ventricular Beats (VEBs) and Supraventricular Premature Beats (SVPBs) to be doubled at a moderate altitude of 1350 meters compared to sea level [49]. Native people who live at high altitudes have higher pulmonary pressure than people who live at sea level; this leads to a very high incidence of Cardiac Cor Pulmonale (CCP) [50]. Furthermore, it has been noted that patients with CCP tend to experience cardiac arrhythmias, particularly supraventricular ones.

Patients with CCP are prone to arrhythmias, particularly supraventricular types, due to severe pulmonary hypertension causing right atrial dilation and atrial remodelling [51-53]. Long-term exposure to a hypoxic environment at high altitudes induces pulmonary vascular remodelling, which leads to Pulmonary Hypertension (PH) and, in extreme cases, even Right Heart Failure (RHF) [54]. A 2019 study showed that individuals living at high altitudes are more likely to be hospitalised for cardiac failure than at sea level [55]. However, an Italian study from 2000 found no significant differences in symptoms, such as arrhythmia, angina, or acute heart failure between individuals with a Left Ventricular Ejection Fraction (LVEF) of 35% living at high altitudes and those at sea level. Nonetheless, it was noted that maximal exercise capacity decreased further at altitude in individuals with low exercise capacity [56]. In the cases of cardiac failure, the presentation was split evenly; one-third of patients showed only diastolic heart failure, another third only systolic heart failure, and the final third exhibited both diastolic and systolic heart failure [54]. Patients with cardiac failure who resided at intermediate-high altitudes experienced more impaired diastolic functions and higher pulmonary pressures, resulting in increased hospitalisations [57]. High altitude also exacerbates decompensation risk in patients with Heart Failure with Reduced Ejection Fraction (HFREF), resulting in more cardiac events and re-hospitalizations [54].

The impact of body shape on physiological responses at high altitudes is an exciting research topic. For instance, a recent study demonstrated that the Modified Haller Index (MHI) effectively assesses chest wall deformity in patients with Idiopathic Pulmonary Fibrosis (IPF), with a concave chest wall (MHI > 2.5) correlating with more severe restrictive lung disease and poorer outcomes. This suggests the MHI could help predict prognosis and guide treatment in such conditions. Exploring how variations in body shape influence respiratory function and adaptation to high-altitude environments presents an interesting avenue for further research [58].

Understanding the complex relationship between high altitude and cardiovascular health is crucial, particularly for individuals with pre-existing heart conditions. Despite the risks, a thorough understanding of the effects of altitude on cardiovascular health is essential for healthcare professionals.

## CONCLUSION

High-altitude exposure presents a multifaceted challenge to cardiovascular health, impacting both acute and chronic conditions. Reduced oxygen levels, barometric pressure, and noticeable temperature changes are characteristics of high altitude that present difficulties for people and cause various physiological reactions. While the majority adapt gradually, some may experience adverse reactions leading to acute altitude illnesses, such as Acute Mountain Sickness (AMS) and, in extreme cases, Chronic Mountain Sickness (CMS). The cardiovascular system's ability to adjust to high altitudes is vital, and failure in adaptation may result in adverse consequences, like arrhythmias, systemic hypertension, and right ventricular failure. The interaction between high altitude and pre-existing cardiovascular conditions is complex. Individuals with coronary artery disease, heart failure, or arrhythmias face increased risks, especially with rapid ascents from lower altitudes. However, studies suggest that stable coronary artery disease patients do not experience a significant increase in adverse outcomes up to 3500 meters.

Conversely, those with unstable angina, recent myocardial infarction, or recent cardiac interventions should avoid high-altitude activities to prevent exacerbation. Notably, high-altitude environments are associated with reduced cardiovascular mortality rates, with studies showing lower mortality from coronary heart disease and stroke at higher altitudes. Additionally, congenital heart disease prevalence is notably higher in newborns and children born at high altitudes, reflecting the severe impact of hypoxia on developing hearts. Further research into the effects of high altitude on cardiovascular health, particularly in individuals with existing conditions, is essential for better risk management. Exploring how body shape influences physiological adaptation to high altitudes could also provide valuable insights, enhancing our understanding of how to mitigate altitude-related health risks effectively.

## LIST OF ABBREVIATIONS

AMS: Acute Mountain Sickness
ASDs: Atrial Septal Defects
BP: Blood Pressure
CAD: Coronary Artery Disease
CCP: Cardiac Cor Pulmonale
CMS: Chronic Mountain Sickness
EPAS1: Endothelial PAS Domain-Containing Protein-1
EPO: Erythropoietin
ET-1: Endothelin-1
HAPE: High-Altitude Pulmonary Edema
HFREF: Heart Failure with Reduced Ejection Fraction
HIF: Hypoxia-Induced Factor
HIF-2α: Hypoxia-Inducible Factor 2α
HPV: Hypoxic Pulmonary Vasoconstriction
HR: Heart rate
PAH: Pulmonary Arterial Hypertension
PDA: Patent Ductus Arteriosus
PH: Pulmonary Hypertension
RHF: Right Heart Failure
RVH: Right Ventricular Hypertrophy
SBP: Systolic Blood Pressure
SMCs: Smooth Muscle Cells
SNPs: Small Nuclear Proteins
SVPB: Supraventricular Premature Beats
VEBs: Ventricular Beats
VSDs: Ventricular Septal Defects
